# Boosting animal performance, immune index and antioxidant status in post-weaned bull calves through dietary augmentation of selective traditional medicinal plants

**DOI:** 10.1016/j.vas.2021.100197

**Published:** 2021-08-18

**Authors:** A.B.M. Rubayet Bostami, M. Rokibul Islam Khan, A.K.M. Zilani Rabbi, M. Nurealam Siddiqui, M. Tofazzal Islam

**Affiliations:** a Department of Animal Science and Nutrition, Faculty of Veterinary Medicine and Animal Science, Bangabandhu Sheikh Mujibur Rahman Agricultural University (BSMRAU), Gazipur-1706, Bangladesh; b Department of Animal Science, Faculty of Animal Husbandry, Bangladesh Agricultural University (BAU), Mymensingh-2202, Bangladesh; c Agricultural Training and Management Development Institute, Dhaka, Bangladesh.; d Department of Biochemistry and Molecular Biology, Bangabandhu Sheikh Mujibur Rahman Agricultural University (BSMRAU), Gazipur-1706, Bangladesh.; e Institute of Biotechnology and Genetic Engineering (IBGE), Bangabandhu Sheikh Mujibur Rahman Agricultural University (BSMRAU), Gazipur-1706, Bangladesh

**Keywords:** Traditional medicinal plant, Feed additives, Growth performance, Blood assay, Immune index, Antioxidant status

## Abstract

Traditional medicinal plants (TMP) are considered valuable alternatives to hazardous synthetic chemical utilized as animal growth promoters. This study aimed to evaluate effects of dietary supplementation of seed and leaf powder (75:25) of three Bangladeshi TMP viz. *Emblica officinalis* Gaertn., *Terminalia bellirica* Gaertn. Roxb., and *Terminalia chebula* Retz. on growth performance, digestibility of nutrients, hematological indices, immune index and antioxidant status in post-weaned bull calves. Dietary treatments: TMP1 (basal diet = green grass + concentrate), TMP2 (basal diet + 0.5% mixture of seed and leaf (75:25) of *E. officinalis* Gaertn.), TMP3 (basal diet + 0.5% mixture of seed and leaf (75:25) of *T. bellirica* Gaertn. Roxb.), and TMP4 (basal diet + 0.5% mixture of seed and leaf (75:25) of *T. chebula* Retz.). Supplementation of TMP significantly (*p < 0.10*) improved weight gain without affecting digestibility. Hematological indices did not change much by treatment of TMP except for enhancement of monocyte (*p* < 0.10) and platelet percentage (*p* < 0.05). A reduction in IGF, enhancement (*p* < 0.05) of lymphocyte proliferation, and IgG were recorded in TMP2, TMP3 and TMP4 compared to TMP1. Decreasing (*p* < 0.05) trend of interleukins-1 and 6, and an elevation of TNF-*α* and antioxidant status were recorded in the TMP2, TMP3, and TMP4 in comparison to TMP1. An elevated level of total antioxidant and glutathione peroxidase activities were found in calves treated with TMP2, TMP3, and TMP4 (*p* < 0.05) relative to TMP1. Taken together, our results indicate that supplementation of feed with Bangladeshi TMP promoted growth and some health indices like immune-advocating efficacy of post-weaned bull calves.

## Highlights

• The effects of dietary supplementation of three Bangladeshi traditional medicinal plants (TMP) were evaluated on growth performance, digestibility of nutrients, hematological indices, immune index and antioxidant status in post-weaned bull calves.
• Feed supplementation with TMP enhanced health indices such as augmentation of monocyte, platelet percentage, IgG and lymphocyte proliferation whereas a reduction of IGF was recorded.
• An elevated level of TNF-α, total antioxidant and glutathione peroxidase activities were found in calves treated with medicinal herbs.

## 1 Introduction

Various factors such as genetics, nutrition, management, pre- and post-slaughter conditions impacts the performance and on meat quality of animals (Bostami, Mun & Yang, 2018; Taberlet, Coissac, Pansu & Pompanon, 2011). To satisfy the increasing demand for animal products due to the rapidly increasing world population, animal production has been intensified. Consequently, for the increase of productivity and quality of animal meat, breeding and nutritional research approaches are also being continued in both developed and developing countries. The intensification of animal production is often limited by both availabilities of inadequate indigenous feed and desired breed of animals in most of the developing countries (Eastridge, 2006; Kamanzi & Mapiye, 2012). Therefore, novel sources of low-cost feed supplementation are needed for ruminant's feed and rumen or digestive system manipulation to maximize ruminant productivity. In the animal industry, feed comprises by far the largest component of input cost. Hence, the efficiency of feed utilization in terms of herd output is the primary consideration in any technological development.

Addition of synthetic growth promoters, antibiotics, anabolic steroids or other chemical substances are the common practices in case of animal production systems for the uplifting of growth and milk production or meat quality enrichment (Courtheyn et al., 2002; Ronquillo & Hernandez, 2017). However, general consumer concern has grown against the use of such synthetic chemical products due to a number of reasons, such as deleterious residual effects on human health, environment, and biodiversity (Courtheyn et al., 2002; Gadde, Kim, Oh & Lillehoj, 2017). Since January 2006, the European Parliament has restricted the use of antibiotics in animal feeds (OJEU, 2003). Consequently, animal scientists are seeking alternative additives to the synthetic chemicals that could improve feed efficiency and animal productivity by favorably modulating metabolism and manipulating the gut functions in both ruminant and non-ruminants (Greathead, 2003; Kamruzzaman, Torita, Sako, Al-Mamun & Sano, 2011). Supplementation of feed with native traditional herbal medicines are considered as promising alternatives to the hazardous synthetic antibiotics and growth promoters for the animal industry (Bodas et al., 2008; Cardozo, Calsamiglia, Ferret & Kamel, 2004; Busquet, Calsamiglia, Ferret, Carro & Kamel, 2005; Islam et al., 2014).

The oriental herbal medicinal plants possess various organic acids, bioactive secondary metabolites such as alkaloids, phenolics, terpenoids, vitamins and minerals that aid digestion and cure gastroenteric disorders, exterminate parasites, constipation, diarrhea, anorexia, and neuralgia (Kim, Kim, Kim, Oh & Jung, 1994; Windisch, Schedle, Plitzner & Kroismayr, 2008). These medicinal herbs have anti-inflammatory, antioxidant, antitussive, aperients, astringent, anti-aging, diuretic, hepatoprotective, laxative, nutritive tonic, digestive aid and neuroprotective activities in animals and humans (Prasanth, Sivamaruthi, Chaiyasut & Tencomnao, 2019; Yang, Chowdhury, Huo & Gong, 2015). Among various medicinal plants in Bangladesh, *Emblica officinalis* (Gaertn.) or *Phyllanthus emblica* Linn. (*Euphorbeaceae*) (amla in Bengali), *Terminalia bellirica* (Gaertn*.*) Roxb. (*Combretaceae*) (bahera in Bengali) and *Terminalia chebula* Retz. (*Combretaceae*) (haritaki in Bengali) are popularly used to treat various human diseases as they contain various bioactive secondary metabolites (saponins, alkaloids, flavonoids etc.), essential oils, organosulphur, micronutrients, hormone-like agents, antimicrobial agents, antioxidants and immune-promoting agents (Hazra, Sarkar, Biswas & Mandal, 2010; Vani, Rajani, Sarkar & Shishoo, 1997). A large body of study pinpointed the presence of bioactive phenolics and other compounds in *E. officinalis, T. bellirica* and *T. chebula* (Bown, 1995; Saxena, Saxena, Nema, Singh & Gupta, 2013) that are attributable in the different functions in the health and physiology of humans and animals with the diverse mode of actions (Belapurkar, Goyal & Tiwari-Barua, 2014; Tewari et al., 2017; Variya, Bakrania & Patel, 2016).

*E. officinalis* Gaertn. is very rich in vitamin C, and other vitamins like vitamin B complex and carotene, and minerals such as calcium, iron and phosphorus. It shows chemo-preventive radio-modulatory, chemo-modulatory effects, free radical scavenging, antioxidant, anti-mutagenic, anti-inflammatory, anti-cancer, and immunomodulatory properties (Baliga & Dsouza, 2011). Dietary augmentation of *E. officinalis* Gaertn. is effective in enhancing productivity and diarrhea prevention in sheep (Bostami, Selim, Hoque, Rabbi & Siddiqui, 2015b). Several lines of evidence suggest that *T. bellirica* Gaertn*.* helps in preventing disease, bestows longevity, improves intellectual prowess, and strength and aids in mental refreshment of humans (Pal & Jain, 1989). The *T. chebula* Retz*.* composed of chebulinic acid, tannic acid, ellagic acid, 2,4-chebulyl-*β*-d-glucopyranose, chebulinic acid and some other phytochemicals. It is used as a purported antitussive, homeostatic, cardiotonic, laxative and diuretic (Saleem, Husheem, Härkönen & Pihlaja, 2002; Tewari et al., 2017).

The biological efficacy of phytogenic feed additives represents a scattered picture in different research findings for human, non-ruminants and ruminants. The majority of experimental results indicate that supplementation of traditional herbal medicines improve feed to gain ratio and positively impact on the health of animals. There exists a wide variation in biological effects induced by phytogenic feed additives reflect the experimental approaches used to test the suitability of these substances as growth-promoting feed additives to animals. Proper selection of the traditional medicinal plants as herbal feed additives from the potential plants, presence of bioactive compounds, and dietary doses are most critical for testing their efficacy on animal production (Bostami et al., 2015a; Bostami, Sarker & Yang, 2017; Islam et al., 2016; Siddiqui, Islam, Sayed & Hossain, 2015). A large body of literature is available on the effects of herbal feed supplement on growth and meat quality of broiler and pigs (Ahmed et al., 2015; Galli et al., 2020; Islam et al., 2014; Loetscher, Kreuzer & Messikommer, 2013; Sarker, Bostami, Kim, Ji & Yang, 2016; Zhu et al., 2019). Very few researches are conducted on plant extracts as rumen moderator in ruminant diets (Kutlu, 2007). There is scant information available on the effects of natural feed supplements on growth, immunity, and antioxidant status of ruminants possibly due to their complex digestive system (Valenzuela-Grijalva, Pinelli-Saavedra, Muhlia-Almazan, Domínguez-Díaz & González-Ríos, 2017). Therefore, it is important to evaluate the effects of supplementation of promising traditional medicinal plants as natural feed additives on growth performance of ruminant animals. Literature of traditional medicinal plants as natural feed additives on post-weaned crossbred (Local × Holstein Friesian) bull or heifer calves’ diet is very limited. Previous studies reported that, supplementation of 0.3%, 0.5% or 0.75% *E. officinalis* fruit powder or pomace exhibited better growth performance, profitability, digestibility, beneficial effects to improve HSP-70 expression during extreme summer, and protect broilers against hemato-biochemical alterations and experimental aflatoxicosis (Dalal, Panwar, Ahlawat, Tewatia & Sheoran, 2018a;2018b; Padmaja, Madhuri, Kumar & Anjaneyulu, 2009; Sapcota, Islam & Upadhyaya, 2006). On the other hand, 0.4 to 0.8% of *E. officinalis* seed powder improve the growth performance and decrease pathogenic *E. coli* and diarrhea prevalence in sheep (Bostami et al., 2015b).

*E. officinalis* Gaertn. or *P. emblica* Linn. (*Euphorbeaceae), T. bellirica* Gaertn. Roxb. (*Combretaceae*) and *T. chebula* Retz. (*Combretaceae*) are the three most famous traditional medicinal plants in Bangladesh and in Indian subcontinent to treat various diseases of humans. It was hypothesized that these locally available traditional medicinal plants positively impact growth, digestibility, hematological indices, immunity, and antioxidant status of post-weaned bull calves. To test this hypothesis, the specific objectives of this study were to evaluate the effect of supplementation of seeds and leaves (75:25) of *E. officinalis, T. bellirica* and *T. chebula* at the dose of 0.5% in feed on growth, nutrient digestibility, hematological indices, immune index, and antioxidant status of crossbred (Local × Holstein Friesian) post-weaned bull claves.

## 2 Materials and methods

### 2.1 Experimental design, animals and diets

The effect of seeds and leaf of three famous traditional medicinal plants viz. *E. officinalis* Gaertn. or *P. emblica* Linn. (*Euphorbeaceae), T. bellirica* Gaertn. Roxb. (*Combretaceae*) and *T. chebula* Retz. (*Combretaceae*) as natural feed additives were evaluated on crossbred (Local × Holstein Friesian) post-weaned bull claves at the Livestock and Poultry Farm, Faculty of Veterinary Medicine and Animal Science, Bangabandhu Sheikh Mujibur Rahman Agricultural University (BSMRAU), Gazipur-1706, Bangladesh. The feeding trial of traditional medicinal plants (TMP) was conducted for a period of 90 days. Twenty-four animals of almost similar age and body weight (about 6 months; body weight 51.0 ± 1.0) were selected and randomly distributed to the control and treatment groups having six calves in each group. The control group received only roughage and concentrate feed without any TMP as feed additives. On the other hand, three treatment groups received different traditional medicinal plants as natural feed additives with the roughage and concentrate feed. The dietary treatment groups were TMP1 (basal diet = green grass + concentrate), TMP2 (basal diet + 0.5% mixture of seed and leaf (75:25) of *E. officinalis* Gaertn.), TMP3 (basal diet + 0.5% mixture of seed and leaf (75:25) of *T. bellirica* Gaertn. Roxb*.*), and TMP4 (basal diet + 0.5% mixture of seed and leaf (75:25) of *T. chebula* Retz.). The experiment was ethically approved by BSMRAU, Bangladesh authority. All the animals were reared according to the animal care and management committee of Bangabandhu Sheikh Mujibur Rahman Agricultural University, Bangladesh, and followed the guidelines and suggestions of Directorate of Livestock Services and Bangladesh Livestock Research Institute of the People's Republic of Bangladesh. Where, ethical issues and animal welfare aspects were applied to confer the animal rights.

### 2.2 Preparation, processing and analysis of samples

The whole seeds and leaves (75:25) of *E. officinalis* Gaertn. or *P. emblica* Linn. (*Euphorbeaceae), T. bellirica* Gaertn. Roxb. (*Combretaceae*) and *T. chebula* Retz. (*Combretaceae*) was dried at 60 °C, ground and stored in air tight containers. All the medicinal plants were collected from BSMRAU campus, Bangladesh that were authenticated by a plant taxonomist Professor Md. Abdul Baset Mia of the Department of Crop Botany of BSMRAU, Bangladesh. The medicinal plant materials were processed and prepared at Animal Science and Nutrition Laboratory, BSMRAU, Bangladesh. Furthermore, processed medicinal plant materials were checked and authenticated by the ethical approval committee of BSMRAU, Bangladesh for using them as feed supplements for the ruminants. Additionally, primary experiments with poultry and ruminants, these medicinal plants did not show any detrimental impacts on health and productivity of the tested animals, rather these materials promoted health indicators (Bostami et al. unpublished; Bostami et al., 2015b, 2021). The whole seeds and leaves (75:25) of traditional medicinal plants as powder form were used as the natural feed additives in the current study.

Proximate analyses (dry matter, crude protein, ether extract and ash) of green grass and concentrates and traditional medicinal plants as natural feed additives were carried out for each sample in triplicate for dry matter (DM), crude protein (CP), Crude fat (EE), nitrogen free extract (NFE) and ash following the AOAC (1990). Official Methods of Analysis. Association of Official Analytical Chemistry (15 Ed), Washington, D.C., U.S.A. While for determination of Ca, P, Na and K concentrations the methods of Jackson (1969) was followed. In addition to that, the determination of Mg concentrations was estimated by following the methods as described by Kalra and Maynard (1991). Moreover, determination of Mn, Cu and Zn was done by following the method describe by McLaren, Swift and Quin (1984)). Table 1 shows the nutrient composition of the experimental diets. The proximate composition of *E. officinalis, T. belerica* and *T. chebula* was presented in Table 2.

**Table 1:** Feed ingredients in the experimental diet of post-weaned crossbred (Local × Holstein Friesian) bull claves.

Ingredients	Percentage (%) as fed-basis
Corn, ground	22.37
Wheat bran	15.00
Wheat, ground	15.00
Rice bran	10.00
Cotton seed meal	7.00
Corn gluten meal	4.50
Protein concentrate	9.90
Sunflower meal	2.50
Soybean meal	2.76
Rapeseed meal	1.00
Distillers dried grains	1.00
Molasses	5.00
Salt	0.60
Limestone (1 mm)	1.87
Di calcium phosphate (DCP)	0.50
Calcium sulfate	0.30
Mineral premix	0.35
Vitamin premix	0.35
**Chemical composition**	
Moisture	12.29
Crude ash	6.10
Crude fat	2.57
Crude fiber	5.71
Crude protein	14.74
NFE	58.71

NFE: Nitrogen free extract.

**Table 2:** Proximate composition of experimental selective traditional medicinal plants provided with basal diet to post-weaned crossbred (Local × Holstein Friesian) bull claves.

Parameters	*Emblica officinalis*	*Terminalia bellirica*	*Terminalia chebula*
% DM (air dry basis)	95.22	94.87	93.12
CP (% as DM basis)	4.89	6.78	4.32
CF (% as DM basis)	9.11	8.77	9.67
NFE (% as DM basis)	77.83	77.75	81.05
EE (% as DM basis)	1.42	1.27	1.25
Ash (% as DM basis)	6.81	5.42	5.12
Ca (ppm)	0.15	0.14	0.10
P (ppm)	9.21	6.42	8.42
Mg (ppm)	0.32	0.39	0.31
K (ppm)	6196.00	6668.00	6616.00
Na (ppm)	183.45	171.23	172.32
Mn (ppm)	209.76	53.33	36.53
Zn (ppm)	39.45	13.52	22.63
Cu (ppm)	6.02	6.23	7.82

DM: dry matter; CP: crude protein; CF: crude fiber; EE:ether extract; NFE: nitrogen free extract.

### 2.3 Management and monitoring of animals

The trial was carried out at Livestock shed, Livestock and Poultry Farm, Faculty of Veterinary Medicine and Animal Science, Bangabandhu Sheikh Mujibur Rahman Agricultural University (BSMRAU), Gazipur-1706, Bangladesh. Where there was set up of separated pens, each of which was equipped with a feeding and watering trough as required for post-weaned bull calves. All pens were located in the same calf house and the calves were randomly allocated based on treatments. The total area per pen was provided 8.5 m^2^. The house was equipped with good ventilation and the bedding materials used in the pens was chopped straw.

Fresh clean drinking water was available for the calves’ ad libitum. Intake of roughage and concentrate were measured separately and refusals were recorded daily. Manure was removed daily to ensure hygienic condition of the animal house. Temperature and humidity in the calf house was monitored regularly. There was sufficient sun light as well as electricity facility for lighting based on necessity of animals. All calves were weighed monthly throughout the experimental period. Blood samples were collected from each group on the last week of the trial and the samples were further analyzed in the Laboratory of Faculty of Veterinary Medicine and Animal Science of BSMRAU, Bangladesh.

### 2.4 Estimation of digestibility of nutrients

The digestibility coefficients were estimated from the fecal samples taken directly from the rectum of the animals from the seventh to the tenth day in each experimental period according to the following distribution: 7th day – 6 a.m. and 2 p.m.; 8th day – 8 a.m. and 4 p.m.; 9th day – 10 a.m. and 6 p.m.; and 10th day – 12 p.m. and 8 p.m. The feces samples were oven dried (60 °C for 72 h) and processed in a Wiley mill (1 mm). Composite samples were elaborated per animal and experimental period. The digestibility coefficients were estimated by the method described earlier by Lazzarini et al. (2009).

### 2.5 Analyses of hematological indices

A veterinary hematology analyzer kit (ProCOUNTTM, Beckon Dickinson Immuno- Cytometry System, USA) was used for analysis of hematocrit indices of the blood samples collected from the control and traditional medicinal plant treated crossbred post-weaned bull calves. After 90 days of the feeding experiment, blood samples of around 10 ml were collected from the jugular vein of 2 calves in each feeding group. After collection of the blood from each experimental group, analyses of red blood cell (RBC) such as number of RBC, hematocrit (HCT), hemoglobin (Hb) and mean cell volume (MCV), and white blood cell (WBC) such as number of WBC, lymphocyte (LYM) count, monocytes (MONO) count and granulocytes (GRA) count. In addition, platelet percent (PCT) and mean platelet volume (MPV) were estimated.

### 2.6 Measurement of IGF and immunity

Blood sample was collected from the jugular vein of selected post-weaned calves of similar weight range. Following collection, the blood samples were centrifuged (10 min, *1000* × *g*), serum was collected, processed and analyzed. Where, serum insulin-like growth factor-I (IGF-I) levels were determined by RIA using ^125^I-labelled IGF-I ([T*hr*^59^]-rhIGF-I; ICN Biomedicals, St Laurent, PQ); where according to the description of Kerr et al. (1990) a monoclonal antibody (MAb) to IGF-I (82–9A) also determined. In a short, the concentration of IGF-I were measured in 50 µl air-dried aliquots of the IGF-I peak (12 mL) obtained by acid chromatography of 0.2 mL serum as described by Zapf, Walter, and Froesch (1981a) Zapf, Walter and Froesch (1981a),1981b) and Daughaday, Yanow and Kapadia (1986). Precipitation of MAb bound ^125^I-labelled IGF-I was performed with rabbit anti-mouse Immunobeads (BioRuO. Richmond. CA.). Immunoglobulins (IgG, IgM and IgA) were measured by using specific Enzyme Linked Immunosorbent Assay (ELISA) kits (Koma Biotech, Seoul, Republic of Korea). The ELISA kits included Bovine IgG ELISA Kit (CAT K3231014, 96well), Bovine IgM ELISA Kit (CAT K3231020, 96well), and Bovine IgA ELISA Kit (CAT K3231012, 96well). The concentrations of immunoglobulins, IgG, IgM and IgA were assayed using appropriately diluted samples by a sandwich ELISA kits and ELISA quantitation kits (Bethyl Laboratories Inc., Montgomery, TX) based on the manufacturer's instructions. Each experiment was run in duplicate and the results represent the means of triplicate experiments. The absorbance of each well at 450 nm was measured within 30 min using a microplate auto-reader (Thermo Lab Systems, Helsinki, Finland). The concentrations of immunoglobulins, IgG, IgM and IgA were determined using standard curves constructed from the respective immunoglobulin standards and the results were expressed as mg/L of serum.

### 2.7 Measurement of cell proliferation assay

A colorimetric assay was used to conduct the Lymphocyte (subtype of white blood cell) proliferation assay with 3-(4, 5-dimethlthiazol-2-yl)−2,5-diphenyltetrazolium bromide (MTT, M-2128, Sigma, St Louis, MO, USA) in cultures of purified peripheral blood mononuclear cells as described previously (Li et al., 2006; Liu et al., 2003). Blood sample was centrifuged at *3000 × g* for 10 min at room temperature in 5 ml of histopaque (density 1.077; Sigma-Aldrich, Dorset, UK) in 10 ml centrifuge tubes. Then the separated cells (approximately 1 ml) were transferred into another 10 ml centrifuge tube containing 4 ml of specific medium 1640 (RPMI; Gibco Life Technologies, New York, NY, USA) and mixed thoroughly. The supernatant was discarded and the pellet was re-suspended in 4 ml of RPMI, following centrifugation at *2000 × g* for 10 min at room temperature. The process was repeated for 2 additional times, then the pellet was re-suspended in 4 ml of RPMI supplemented with penicillin G (100 U/ml; Merck KGaA, Darmstadt, Germany), streptomycin (100 µg/ml; Sigma St Louis, MO, USA), and 10% heat-inactivated newborn calf serum (HyClone Laboratories Inc., Logan, UT, USA). The samples were counted and plated into polypropylene 96-well culture dishes (Costar Corp., Cambridge, MA) at 2 × 10^6^ cells/ml.

Lymphocyte mitogen concanavalin A (Type IV, C-2010, Sigma St Louis, MO, USA) was added at a final concentration of 16 mg/ml of culture medium. All the plates were incubated at 37 °C in an incubator in an atmosphere of 5% CO_2_ (Heraeus, Hongkong, China) for 66 h. The plates were further incubated at 37 °C for 6 h, after the addition of 10 µl of MTT solution (5 mg of MTT/ml in phosphate-buffered saline (0.07 M, pH 7.6) to each well. To lyse the cells and solubilize the MTT crystals, a 100 µl of 10% sodium dodecyl sulfate (Sigma St Louis, MO, USA) in 0.04 M HCl was added. By using an automated microplate reader (Model 550, BioRAD Laboratories Inc., Hercules, CA), the absorbance of all the plates were read at 570 nm. Lymphocyte proliferation was expressed as a proliferation index, which was calculated as the absorbance of wells incubated with concanavalin A divided by the absorbance of wells incubated without addition of concanavalin A.

### 2.8 Measurement of cytokines and antioxidant indices in plasma

Plasma concentrations of interleukin-1, interleukin-6, tumor necrosis factor-*α* and insulin-like growth factor-I were quantified using an enzyme-linked immunosorbent assay (R&D, Minneapolis, MN, USA) according to the manufacturer's instructions. A duplicate counting of assays were performed using TEACAN Plate Reader (TEACAN Asia, Shanghai, China). Plasma albumin and globulin level were measured with an Automatic Biochemical Analyzer (RA-1000; Bayer Corp., Tarrytown, NY, USA) following colorimetric methods and instructions from the manufacturer's corresponding reagent kit (Zhongsheng Biochemical Company, Beijing, China). The total antioxidant capacity, glutathione peroxidase, superoxide dismutase and malondialdehyde levels of plasma assay were conducted using spectrophotometric (Leng Guang SFZ1606017568, Shanghai, China) methods following the instructions of the manufacturer (Nanjing Jiancheng Bioengineering Institute, Nanjing, China) of the kit.

### 2.9 Statistical analyses

All data were subjected to ANOVA using the General Linear Models (GLM) function of the Statistical Analysis System (SAS, 2003, Version 9.1, SAS Institute, Cary, NC, USA). Each experimental pen was considered as the experimental unit for growth performance and digestibility, whereas an individual crossbred animal was served as the experimental unit for blood and plasma assays. Level of significance was considered as *p* < 0.05 where tendency was considered as *p* < 0.10.

## 3 Results

### 3.1 Nutrient composition of the diets, growth response, and digestibility of post-weaned bull claves

The chemical composition of concentrate diet and traditional medicinal plants (TMP): *E. officinalis* Gaertn., *T. bellirica* Gaertn. Roxb. and *T. chebula* Retz. is shown in Tables 1 and 2. Table 3 presents the growth response of crossbred (Local × Holstein Friesian) post-weaned bull claves as supplemented by with (TMP2, TMP3, and TMP4) or without (TMP1) traditional medicinal plants as natural feed additives. The traditional medicinal plants as natural feed additives (TMP2, TMP3, and TMP4) swayed in the tendency of boosting in the final weight and average daily weight gain in the crossbred (Local x Holstein Friesian) post-weaned claves as compared to the untreated control group (TMP1). Among the traditional medicinal plants’ incorporated group, the TMP3 group exhibited higher tendency in final weight and average daily weight gain in relation to TMP1 (*p* < 0.10). However, treatment with traditional medicinal plants did not show any effect on the total tract digestibility of different nutrients in the claves (Table 4). Table 4 shows that the DM, OM and ash digestibility did not significantly (*p* > 0.05) vary in TMP2, TMP3, and TMP4 as compared to TMP1.

**Table 3:** Growth performance of post-weaned crossbred (Local × Holstein Friesian) bull claves supplemented with selective traditional medicinal plants.

Parameters	TMP1	TMP2	TMP3	TMP4	SEM	P-value
Growth						
ILW (kg/calf)	51.76	51.78	51.72	51.64	0.604	0.999
FLW (kg/calf)	72.39^b^	74.77^ab^	76.33^a^	74.60^ab^	0.775	0.054
ADG (kg/d/animal)	0.23^b^	0.26^ab^	0.27^a^	0.26^ab^	0.009	0.110
ADFI (kg/d/animal)	1.51	1.55	1.57	1.56	0.025	0.382
FE (gain/feed)	0.15	0.16	0.17	0.16	0.008	0.414

^a,b,c^ Means in the same row with different superscripts are significantly different (*p*<0.05).

The dietary treatment groups were TMP1 (basal diet = green grass + concentrate), TMP2 (basal diet + 0.5% mixture of seed and leaf (75:25) of *E. officinalis* Gaertn.), TMP3 (basal diet + 0.5% mixture of seed and leaf (75:25) of *T. bellirica* Gaertn. Roxb*.*), and TMP4 (basal diet + 0.5% mixture of seed and leaf (75:25) of *T. chebula* Retz.).

TMP: Traditional medicinal plant, ILW: Initial live weight, FLW: Final live weight; ADG: Average daily weight gain; ADFI: Average daily feed intake; FE: Feed efficiency

**Table 4:** Nutrient digestibility of post-weaned crossbred (Local × Holstein Friesian) bull claves supplemented with selective traditional medicinal plants.

Parameters	TMP1	TMP2	TMP3	TMP4	SEM	*P*-value
Digestibility%						
DM	63.99	63.99	64.64	64.40	0.29	0.366
OM	61.30	61.37	61.59	61.78	0.16	0.395
Ash	75.32	75.90	75.36	75.48	0.29	0.493

^a,b^Means in the same row with different superscripts are significantly different (*p* < 0.05).

The dietary treatment groups were TMP1 (basal diet = green grass + concentrate), TMP2 (basal diet + 0.5% mixture of seed and leaf (75:25) of *E. officinalis* Gaertn.), TMP3 (basal diet + 0.5% mixture of seed and leaf (75:25) of *T. bellirica* Gaertn. Roxb*.*), and TMP4 (basal diet + 0.5% mixture of seed and leaf (75:25) of *T. chebula* Retz.).

TMP: Traditional medicinal plant, DM: dry matter; OM: organic matter.

### 3.2 Hematological indices of post-weaned bull claves

To assess whether the inclusion of traditional medicinal plants has any effects on hematological indices of the calves, we analyzed blood samples of treated and untreated animals (Table 5). Although no significant effects on red blood cells (RBC) and their components, lymphocytes, and granulocytes among the test diets (*p* *>* 0.05) were recorded. However, the tendency of higher values of monocytes was recorded in calves treated with all traditional medicinal plants supplemented groups (TMP2, TMP3, and TMP4) compared to control (TMP1) group (*p* *<* 0.05), where the higher value of platelet percentage was found in traditional medicinal plants supplemented group (TMP2, TMP3, and TMP4) compared to control (TMP1) group (*p* *<* 0.05).

**Table 5:** Hematological indices of post-weaned crossbred (Local × Holstein Friesian) bull claves supplemented with selective traditional medicinal plants.

Parameters	TMP1	TMP2	TMP3	TMP4	SEM	*P*-value
Hematological indices						
Red Blood Cells						
RBC (10^6^/mm^3^)	14.03	14.24	14.23	14.00	0.37	0.9454
HGB (g/L)	121.75	125.80	123.95	119.25	3.04	0.5069
HCT (%)	36.47	37.62	37.19	35.53	1.25	0.6868
MCV (fl)	26.00	26.45	26.10	25.35	0.62	0.6627
White Blood Cells						
WBC (10^3^/mm3)	10.72	12.11	11.85	9.77	0.99	0.4116
LYM (10^3^/mm3)	3.93	4.10	4.13	4.21	0.28	0.9119
MONO (10^3^/mm3)	0.53^b^	0.69^a^	0.69^a^	0.68^a^	0.04	0.0618
GRA (10^3^/mm3)	6.25	6.71	6.82	6.71	0.72	0.9510
Platelets						
PCT	0.30^b^	0.49^a^	0.44^a^	0.42^a^	0.03	0.0133
MPV (fl)	5.35	5.21	5.29	5.30	0.13	0.9042

^a, b^Means in the same row with different superscripts are significantly different (*p* < 0.05).

The dietary treatment groups were TMP1 (basal diet = green grass  + concentrate), TMP2 (basal diet + 0.5% mixture of seed and leaf (75:25) of *E. officinalis* Gaertn.), TMP3 (basal diet + 0.5% mixture of seed and leaf (75:25) of *T. bellirica* Gaertn. Roxb*.*), and TMP4 (basal diet + 0.5% mixture of seed and leaf (75:25) of *T. chebula* Retz.).

TMP: Traditional medicinal plant, RBC = Red Blood Cell/Erythrocytes count, HGB = Hemoglobin, HCT = Hematocrit, MCV = Mean cell volume, WBC = White blood cell count, LYM = Lymphocyte count, MONO = Monocytes count, GRA = Granulocytes count, PCT = Platelet percent, MPV = Mean platelet volume.

### 3.3 Blood assay for IGF and immune index of post-weaned bull claves

To understand the immune response of the post-weaned bull calves by the treatment of traditional medicinal plants as natural feed additives, we performed a blood assay for insulin-like growth factor (IGF) and immune index (Table 6). Interestingly, the level of immunoglobulin G (IgG) was significantly (*p* *<* 0.05) elevated in the TMP2, TMP3, and TMP4 treated calves compared to the untreated control (TMP1). Similarly, lymphocyte proliferation was also upgraded significantly (*p* *<* 0.05) in the TMP2, TMP3, and TMP4 treated calves compared to control (TMP1). However, traditional medicinal plants supplementation slightly suppressed the values of insulin-like growth factor-1 (IGF-1) in post-weaned bull calves.

**Table 6:** Plasma IGF and immune index of post-weaned crossbred (Local × Holstein Friesian) bull claves supplemented with selective traditional medicinal plants.

Parameters	TMP1	TMP2	TMP3	TMP4	SEM	*P*-value
IGF and immune index						
IGF-I (µg/L)	86.50^a^	77.46^b^	78.29^b^	76.71^b^	2.29	0.0269
LCP	1.27^b^	1.48^a^	1.52^a^	1.49^a^	0.04	0.0013
Albumin (g/L)	25.07	23.89	24.79	23.55	0.92	0.6212
IgA (mg/L)	43.39	45.25	46.62	45.10	1.64	0.6036
IgM (mg/L)	253.61	257.72	260.17	259.89	2.99	0.4118
IgG (mg/L)	51.49^b^	59.49^a^	60.35^a^	58.38^a^	2.13	0.0401

^a, b^Means in the same row with different superscripts are significantly different (*p* < 0.05).

The dietary treatment groups were TMP1 (basal diet = green grass + concentrate), TMP2 (basal diet + 0.5% mixture of seed and leaf (75:25) of *E. officinalis* Gaertn.), TMP3 (basal diet + 0.5% mixture of seed and leaf (75:25) of *T. bellirica* Gaertn. Roxb*.*), and TMP4 (basal diet + 0.5% mixture of seed and leaf (75:25) of *T. chebula* Retz.).

TMP: Traditional medicinal plant, IGF: Insulin-like growth factor; LCP: Lymphocyte proliferation; IgA: Immunoglobulin A, IgG: Immunoglobulin G, IgM: Immunoglobulin M.

### 3.4 Plasma cytokines and antioxidant status of post-weaned bull claves

**Fig. 1:**
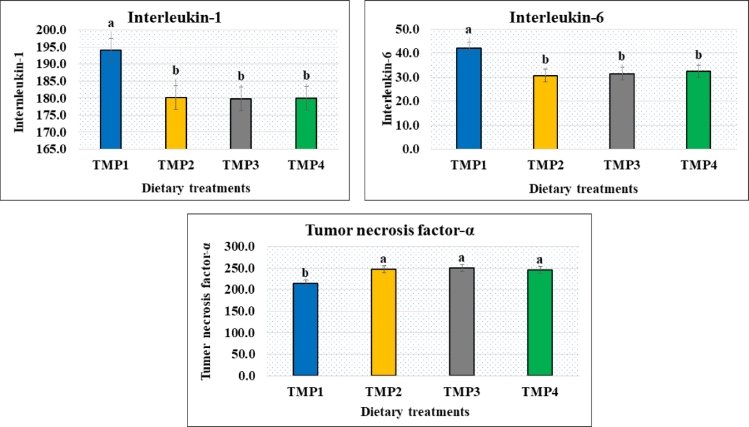
Plasma cytokines (ng/L) of post-weaning crossbred (Local × Holstein Friesian) bull claves supplemented with selective traditional medicinal plants. ^a, b^ Means in the same row with different superscripts are significantly different (*p* < 0.05). The dietary treatment groups were TMP1 (basal diet  = green grass + concentrate), TMP2 (basal diet + 0.5% mixture of seed and leaf (75:25) of *E. officinalis* Gaertn.), TMP3 (basal diet + 0.5% mixture of seed and leaf (75:25) of *T. bellirica* Gaertn. Roxb*.*), and TMP4 (basal diet + 0.5% mixture of seed and leaf (75:25) of *T. chebula* Retz.). TMP: Traditional medicinal plant, TNF- α: Tumor necrosis factor-*α*.

**Fig. 2:**
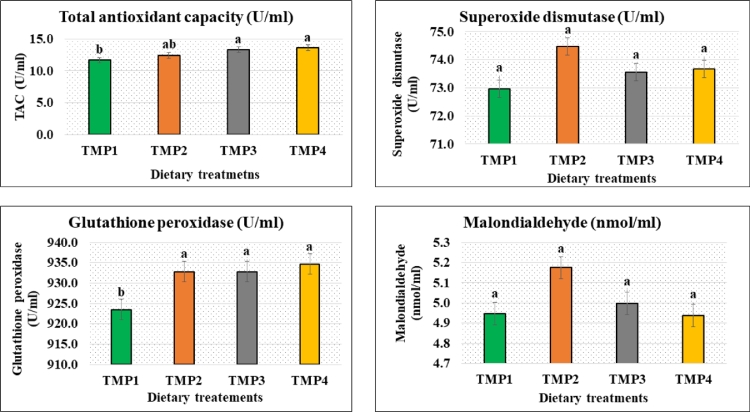
Plasma antioxidant status of post-weaning crossbred (Local × Holstein Friesian) bull claves supplemented with selective traditional medicinal plants. ^a, b^ Means in the same row with different superscripts are significantly different (*p* < 0.05). The dietary treatment groups were TMP1 (basal diet = green grass + concentrate), TMP2 (basal diet + 0.5% mixture of seed and leaf (75:25) of *E. officinalis* Gaertn.), TMP3 (basal diet + 0.5% mixture of seed and leaf (75:25) of *T. bellirica* Gaertn. Roxb*.*), and TMP4 (basal diet + 0.5% mixture of seed and leaf (75:25) of *T. chebula* Retz.). TMP: Traditional medicinal plant, TAC: Total Antioxidant Capacity.

The plasma cytokines and antioxidants data are presented in the Fig. 1, Fig. 2, respectively. Both tumor necrosis factor-α (TNF-*α*) and antioxidant status in post-weaned bull calves increased by the supplementation of feed with traditional medicinal plants. Total antioxidant and glutathione peroxidase activities were significantly (*p* *<* 0.05) increased in the calves fed traditional medicinal plants supplemented feed (TMP2, TMP3, and TMP4) compared to control (TMP1). However, cytokines data indicated that Interleukin-1 and Interleukin-6 diminished (*p* *<* 0.05) in the TMP2, TMP3, and TMP4 treated calves in relation to control (TMP1).

## 4 Discussion

### 4.1 Selective medicinal plants aided growth but unaffected the nutrient digestibility of post-weaned bull calves

The selective medicinal plants as natural feed additives are thought to be as effective alternatives to the hazardous synthetic growth promoters in the animal industry (Bostami et al., 2015a, 2017; Islam et al., 2014; Kumar, Kumar, Roy, Kushwaha & Vaiswani, 2014; Siddiqui et al., 2015). In the current study, we found that feed supplementation with three locally famous medicinal plants viz. *E. officinalis* Gaertn. or *P. emblica* Linn. (*Euphorbeaceae), T. bellirica* Gaertn. Roxb. (*Combretaceae*) and *T. chebula* Retz. (*Combretaceae*) improved the final live weight and average daily weight gain of the crossbred bull calves without affecting their general nutrient digestibility. Weight gain of animals by the supplementation of herbal medicines has previously been reported by several researchers (Sultana et al., 2012; Wang et al., 2011). For instance, a significant weight gain (27%) of the post-weaned bull calf by the feeding additives of an herb, *S. mukorossi* has been demonstrated (Sultana et al., 2012). Similarly, Wang et al. (2011) also observed weight gain in pigs by the feed supplementation with garlic.

The data generated from this study are not enough to elucidate the mode of action of growth promotion of calves by traditional medicinal plants, however, diverse secondary metabolites and biochemical contents of the crude leaf and seed powder might play the beneficial impact likely through the improvement of the ecosystem of gastrointestinal microbiota by suppressing potential pathogenic microorganisms, and improvement of the metabolism of the calves (Bostami et al., 2021; Roth & Kirchgessner, 1998; Windisch et al., 2008). Better final weight and average daily weight gain tendency in the traditional medicinal plants treated groups shown in the present study might be linked with rich presence of phytochemicals such as ellagic acid, ellagitannin, gallic acid, chebulic acid, belleric acid in the seeds and leaves of *E. officinalis* Gaertn., *T. bellirica* Gaertn. Roxb. and *T. chebula* Retz. (*Combretaceae*) (Bown, 1995; Saxena et al., 2013; Tewari et al., 2017). It has been observed that bioactive secondary metabolites, essential oils and organic acids present in the herbal feed additives modulate the gut microbial environment to exert beneficial interactions (Valenzuela-Grijalva et al., 2017); consequently, enhance the growth performance of the treated animals (Partanen & Mroz, 1999).

Although medicinal principles of three Bangladeshi traditional medicinal plants as natural feed additives used in this study have been reported in the literature (Bown, 1995; Saxena et al., 2013), positive effect of 0.6% *E. officinalis* in sheep has been reported (Bostami et al., 2015b). In the current study, we demonstrated the improvement of the growth of crossbred weaned calves by supplementation of feed with 0.5% seed and leaf powders (75:25) of these native plants. Among the traditional medicinal plants, supplementation of the seed and leaf powder of *T. bellirica* Roxb. showed the best performances. The main active phytoconstituents of medicinal importance of *T. bellirica* Roxb. are ellagic acid, ethylgalate, gallyl glucose, glucoside, tannins, chebulanic acid, gallic acid etc. that may have antidiarrheal, antimicrobial activity, antidiabetic, antisecretory, analgesic, antihypertensive, antipyretic, hepatoprotective, anticancer, antidepressant-like, anti-urolithiatic, antioxidant, antiulcer, angiogenesis, antipsychotic, and anti-disorder activities (Jayesh, Karishma, Vysakh, Gopika & Latha, 2020; Kumar & Khurana, 2018). The phytochemicals in *T. bellirica* Roxb*.* are responsible for many pharmacological activities that might be attributable in the higher final body weight and average daily weight gain of the calves. Further research is needed for isolation of specific bioactive compound(s) involved in growth promoting effect of *T. bellirica* Roxb*.* on calves.

Digestibility of feed materials in livestock and poultry is important for better feed efficiency and productivity. Supplementation of feed with peppermint increases nutrient digestibility (Ando, Nishida, Ishida, Hosoda & Bayaru, 2003), increase in ruminal DM and OM digestibility after feeding garlic oil to dairy cattle (Kongmun, Wanapat, Pakdee & Navanukraw, 2010; Yang et al., 2007) and improvement of dry matter and nitrogen digestibility of animals by the plant essential oils have been demonstrated (Cho et al., 2005; Zhang, Jung, Kim, Kim & Kim, 2012). However, inconsistent to previous results, in our study, supplementation of leaf and seed powder of three traditional medicinal plants did not show any effect on nutrient digestibility of calves compared to the control.

### 4.2 Selective medicinal plants on blood hematological indices of post-weaned bull calves

Analyses of hematological indices of blood revealed that supplementation of three traditional medicinal plants significantly enhanced monocyte (*p* < 0.10) and platelet percentage (*p* < 0.05). Interestingly, a significantly higher value of platelets was recorded in the blood of calves treated with leaf and seed powder of *T. bellirica* Gaertn. Roxb (TMP3) compared to the untreated control (TMP1). A tendency of higher monocytes found in TMP2, TMP3, and TMP4 groups compared to TMP1 group, where TMP3 showed the highest value. The results of the present study indicate a better tendency in monocytic action in the cellular mechanism in the case of TMP2, TMP3, and TMP4 groups. Platelets or thrombocytes are generally tiny disk-shaped cells, which help in prevention of abnormal or excessive bleeding by forming clots. They are much smaller in size than other types of blood cells. They group together to form clumps, or a plug, in the hole of a vessel to stop bleeding. A deficiency of platelets can cause bleeding of the mucous membranes or other tissues, such as the skin. The higher value of platelets found in the present study might be beneficial to the physiology or pharmacology of crossbred post-weaned calves due to inclusion of natural feed additives in their diet. A positive influence in broiler hematological profile and immune parameters by supplementing phytochemicals, genistein and hesperidin has been reported (Kamboh et al., 2018). Therefore, the phytochemicals such as flavonoids present in the traditional medicinal plants *E. officinalis, T. bellirica* and *T. chebula* might play some positive roles in the pharmacological indices in the crossbred post-weaned bull calves in this study.

Except monocytes and platelets, other blood hematological indices data remained almost unchanged by the application of traditional medicinal plants as natural feed additives in the calves. The red blood cells are produced into the bone marrow, and are continuously being formed and broken down. The life span of the cell is approximately 120 days in the circulatory system and is eventually removed by the spleen, and is composed of hemoglobin, an iron-rich protein which usually carry or picks up the oxygen as the blood passes through lungs, and transports and releases it to organs and tissues throughout the body. A non-significant impact of traditional medicinal plants on RBC of calves found in this study indicates no negative impact or risk of anemia in the calves’ body physiology with herbal applications. Similarly, the data of WBC and its other components such as lymphocyte and granulocyte did not differ significantly among the dietary groups. No significant differences on RBC and their components found in this study suggest that there were no negative impacts of the addition of traditional medicinal plants as herbal feed additives in case of utilizing them in the diet of crossbred post-weaned bull calves.

### 4.3 Selective medicinal plants ameliorate the IGF and immune index of post-weaned bull calves

In the current study, irrespective of traditional medicinal plants (TMP2, TMP3, and TMP4) used, the herbal feed additives significantly increased IGF-I compared to untreated control (TMP1) (Table 6). This finding partially supportive to the development of final weight and daily weight gain of the crossbred post-weaned calves by the supplementation of traditional medicinal plants. Interestingly, a significant enhancement of lymphocyte proliferation was observed in the traditional medicinal plants treated groups, TMP2, TMP3 and TMP4 compared to the control group (TMP1). A significant uplifting of lymphocyte proliferation has previously been reported by feeding essential oil extracted from medicinal plants (Li et al., 2012). In our study, flavonoids and phytochemicals present in the *E. officinalis, T. bellirica* and *T. chebula* might be involved in the modification of cellular proliferation. Exposure of animals to flavonoids rich materials can affect lymphocyte proliferation and activation induced cell death (AICD) and modulation of cytokines through suppression of Th1 cytokine IL-2 production can affect proliferative responses (Maslowski & Mackay, 2011; Ramiro-Puig & Castell, 2009). Kaempferol prevents proliferation in the mixed lymphocyte culture (MLC) in vitro in a dose-dependent manner (Chen & Chen, 2013; Maslowski & Mackay, 2011; Najera et al., 2001; Zhang, Zhang, Ji, Wang & Qian, 2000). Down regulation of IL-2 secretion and IL-2 receptor expression by cocoa extract has been reported to reduce lymphocyte proliferation (Ramiro-Puig & Castell, 2009).

Genetic potentials as well as nutritional aspects greatly impacted on the disease prevention capability and cellular immunity in both human and animals. Growth-promoting feed additives relieve the host animals from immune defense stress during critical situations and increase the intestinal availability of essential nutrients for absorption, thereby helping animals to show a better performance within the framework of their genetic potential (Windisch et al., 2008). The nutritional status is reflected by the plasma insulin-like growth factor (IGF) (Ketelslegers, Maiter, Maes, Underwood & Thissen, 1995). There can be a positive correlation between plasma IGF and growth promotion in the case animals (Owens, Hinds & Soderlund, 1999).

Immunoglobulin G (IgG) is indicative of an individual's immune status to particular pathogens. It is well known that IgG is a type of antibody which is created and released by plasma B cells in the blood and extracellular fluid. Immunoglobulin G also plays vital function in the antibody-dependent cell-mediated cytotoxicity and intracellular antibody-mediated proteolysis (Mallery et al., 2010). One of the interesting findings in the present study was that application of traditional medicinal plants (TMP2, TMP3, and TMP4) significantly improved the immunoglobulin G in treated calves compared to the untreated control TMP1. The traditional medicinal plants used in this study were seed and leaf powder of *E. officinalis* (TMP2)*, T. belerica* (TMP3) and *T. chebula* (TMP4). Although mechanisms of IgG enhancement are not known in the present study, secondary metabolites in the leaf and seed powder of *E. officinalis, T. belerica and T. chebula* might act on cellular activity, viability and defensive function in case of traditional medicinal plants treated calves. A further study is needed to test these hypotheses.

Saleem et al. (2002) demonstrated that *T. chebula* extracts inhibit cell proliferation, suppress the cell viability, and induce cell death. Flow cytometry and other analyses of *T. chebula* extract exhibit apoptosis at a lower concentrations, whereas, necrosis attributes the major mechanism of cell death at higher concentrations (Saleem et al., 2002). Similarly, Eucalyptus oil extracts trigger innate cell-mediated immune responses in rats through activation of the macrophages and elevation of the phagocytic ability coupled with a lower release of IL-4, IL-6 and TNF-α pro-inflammatory cytokines (Serafino et al., 2008). However, no positive impact of essential oils or plant extracts on immune responses has been reported (Li et al., 2012).

### 4.4 Selective medicinal plants escalated the plasma cytokines and antioxidant status of post-weaned bull calves

There are three major cytokines: interleukin-1, interleukin-6, and tumor necrosis factor-*α* that play important role in cellular immunity (Johnson, 1997). Higher levels of cytokines like interleukin-6 influence tissue damage (Pié et al., 2004), suppress growth hormone secretion (Johnson, 1997), and alter the energy and protein metabolism to negatively impact the performance of the animal (Spurlock, 1997). In the present study, both interleukin-1 and interleukin-6 down trended by the treatment of traditional medicinal plants in post-weaning calves compared to untreated control (TMP1). Reduction of interleukin-6 in pigs by the feeding treatment of essential oil extracted from medicinal herbs has been reported (Li et al., 2012). Decrease in plasma interleukin-1*β* and increase in lymphocyte proliferation by dietary *Astragalus membranaceus glucan* has also been described by Mao et al. (2005) and Li et al. (2012) reported a decrement of interleukin-6 influences in the improvement of growth response. In the present study, we also observed the growth promotion in calves with the lower value of interleukin-6 in the traditional medicinal plants treated groups (TMP2, TMP3 and TMP4) compared to control (TMP1).

One of the important findings of the current study was that tumor necrosis factor-α expanded in the calves treated with traditional medicinal plants as herbal feed additives (TMP2, TMP3 and TMP4) compared to untreated control (TMP1). Enhancement of immune responses of animals by herbal treatments has previously been reported (Kong et al., 2007; Yin et al., 2008). Medicinal herbs or their extracts are well known for their antioxidative properties (Graßmann, Schneider, Weiser & Elstner, 2001; Wei & Shibamoto, 2007). Non-enzymatic antioxidant defense is marked by plasma total antioxidant capacity (Wang et al., 2008). Elevation of total antioxidant capacity in animals by the addition of dietary essential oils has been reported (Li et al., 2012). An important finding of this study was that a significant higher total antioxidant capacity in the traditional medicinal plant fed groups of calves (TMP2, TMP3 and TMP3) compared to untreated control group (TMP1) might be indicative to lower endogenous lipid peroxidation by the secondary metabolites of the used herbs. The main biological function of glutathione peroxidase (GPx) is to protect the organism from oxidative damage, where GPx is the general name of an enzyme family having peroxidase activity. The biochemical role of GPx is to reduce lipid hydroperoxides to their corresponding alcohols and to suppress free hydrogen peroxide to water. Glutathione peroxidase depends on the micronutrient selenium (Se), plays a captious role in the diminishing of lipid and hydrogen peroxides (Arthur, 2001).

With decrement of GPx activity, more hydrogen peroxide can present, which can result direct tissue damage and activation of nuclear factor-κB–related inflammatory pathways (Meyer, Pahl & Baeuerle, 1994; Yu & Chung, 2006). In the present study, traditional medicinal plants enhanced the GPx activity in calves compared to untreated control. It indicates a positive impact on the reduction of lipid oxidation through balancing the free radicals. The antioxidant system comprises extrinsic antioxidant nutrients and intrinsic enzymes, which aids to decline free radicals to less toxic states (Espinoza et al., 2008; Vertuani, Angusti & Manfredini, 2004). Therefore, traditional medicinal plants as herbal feed additives in the diet of crossbred post-weaned bull calves showed a positive impact on antioxidant status compared to untreated group. The antioxidative properties of seeds and leaves of *E. officinalis* Gaertn., *T. bellirica* Gaertn. Roxb. and *T. chebula* Retz. might played a significant role on influencing the total antioxidant capacity and GPx in treated calves that consequently resulted in better cellular immunity in the animals.

## 5 Conclusion

In conclusion, supplementation of feed with the three traditional medicinal plants (seed:leaf at the ratio of 75:25) of Bangladesh, *E. officinalis* Gaertn., *T. bellirica* Gaertn. Roxb. and *T. chebula* Retz. significantly improved growth (final live weight and average daily weight gain) without affecting digestibility in crossbred (Local × Holstein Friesian) post-weaned bull calves. Although most of the hematological indices remained unaffected, supplementation of traditional medicinal plants increased monocytes and platelet percentage in the blood. Traditional medicinal plants as herbal feed additives also positively regulated the plasma assay and antioxidant status of the calves. Furthermore, lymphocyte proliferation, IgG, and TNF-*α* were also improved but insulin-like growth factor and interleukins-1 and 6 were suppressed. Taken together, these results suggest that supplementation of a low dose (0.5% in feed) of locally available traditional medicinal plants, *E. officinalis* Gaertn., *T. bellirica* Gaertn. Roxb. and *T. chebula* Retz. (seed:leaf at the ratio of 75:25) could be used as natural feed additives alternative to hazardous synthetic chemical growth promoter. Further studies are needed to determine the appropriate doses, and elucidate the mechanisms of growth promoting and immunomodulating effects of these traditional medicinal plants prior to their practical application in the animal industry as a low cost and eco-friendly feed additives.

## Funding

The research was conducted by the financial support of the Research and Management Committee of BSMRAU, Gazipur, and the Ministry of Science and Technology, Bangladesh.

## Declaration of Competing Interest

Authors are declaring no conflict of interest regarding this research and publications.

## Appendix Supplementary materials

Image, application 1
