# Supplementary material for: Boosting animal performance, immune index and antioxidant status in post-weaned bull calves through dietary augmentation of selective traditional medicinal plants
Source: Vet Anim Sci. 2021 Aug 18;14:100197. doi: 10.1016/j.vas.2021.100197 (PMC8426563; doi:10.1016/j.vas.2021.100197)
Supplement: Supplementary file 1 [file mmc1.pdf]

[Annex no. 2]

Animal Test Plan

Reception Number		BSMRAU/IBGE/ERC/010	
Animal Test Name		Boosting animal performance, immune index and antioxidant status in post-weaned bull calves through dietary augmentation of selective traditional medicinal plants	
Director of Research (Animal Experiments)		Dr. A.B.M. Rubayet Bostami, Department of Animal Science and Nutrition, FVMAS, BSMRAU, Gazipur-1706, Bangladesh	
Duration of the experiment		After BSMRAU/IBGE/ERC approval ~ 2020. 06. 30	
Approval number	BSMRAU/IBGE/ERC/010	Approval Date	2019.07.15
Remarks		The head of the study (animal experiment) will follow the following compliance requirements and<br>Please submit your termination report [see attachment file] within 3 months of termination.	

Attached: Animal Test End Report (Form) Part 1.

The Committee approves the above animal test plans.

Approval Date	2019.07.15

Chairman, Ethical Review Committee, Institute of Biotechnology and Genetic Engineering, Bangabandhu Sheikh Mujibur Rahman Agricultural University, Gazipur-1706, Bangladesh

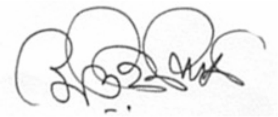

Professor Md. Tofazzal Islam
e-mail: tofazzalislam@bsmrau.edu.bd
Telephone: +880-1714001414

※ Animal test officers are advised to comply with the following:

1. Research should be conducted in accordance with the approved animal test plan approval application form, and when requested by the Committee, a report related to the progress of the research shall be submitted to the Animal Experiment Ethics Committee of BSMRAU.
2. In the event that field inspections are conducted at the level of investigation and supervision of the tasks deliberated by the Committee, the experimental officer shall prepare and cooperate with the documents related to the conduct of the research in an appropriate way in the event of a smooth inspection section.
3. The test supervisor shall conduct his/her own training on hygiene and safety to the animal experimenter before the animal experiment, clean the breeding environment (place) during the experiment to prevent us from causing damage due to infection, and consign the private body treatment to the designated professional company, and please make sure that the treatment is not in violation of the relevant laws and regulations.
4. Receive expert guidance (advice) on animal experiments so that the damage to animals can be minimized, and if necessary in accordance with relevant laws and procedures, please obtain

approval from the relevant authorities, such as the Centers for Disease Control, before animal experiments.

5. Approval of animal test plans cannot be used for advertising (publicity), the end report [see ad-on form below] must be submitted to the committee within 3 months after the end of the animal experiment, and records related to the experiment should be kept for at least 5 years as of the end of the experiment.
